# Exotic *Halophila stipulacea* is an introduced carbon sink for the Eastern Mediterranean Sea

**DOI:** 10.1038/s41598-019-45046-w

**Published:** 2019-07-03

**Authors:** Eugenia T. Apostolaki, Salvatrice Vizzini, Veronica Santinelli, Helen Kaberi, Cristina Andolina, Evangelos Papathanassiou

**Affiliations:** 1 Institute of Oceanography, Hellenic Center for Marine Research, PO Box 2214, 71003 Heraklion, Crete Greece; 2 Department of Earth and Marine Sciences, University of Palermo, Via Archirafi 18, 90123 Palermo, Italy; 3 CoNISMa, Consorzio Nazionale Interuniversitario per le Scienze del Mare, Piazzale Flaminio 9, 00196 Roma, Italy; 4 Institute of Oceanography, Hellenic Centre for Marine Research, PO Box 712, 19013 Anavyssos, Attiki Greece

**Keywords:** Carbon cycle, Element cycles, Invasive species, Plant ecology, Marine biology

## Abstract

Carbon and nitrogen storage in exotic *Halophila stipulacea* were compared to that in native *Posidonia oceanica* and *Cymodocea nodosa* meadows and adjacent unvegetated sediments of the Eastern Mediterranean Sea and to that in native *H. stipulacea* of the Red Sea at sites with different biogeochemical conditions and level of human pressure. Exotic *H. stipulacea* possessed considerable storing capacity, with 2-fold higher C_org_ stock (0.71 ± 0.05 kg m^−2^ in the top 20 cm of sediment) and burial (14.78 gC_org_ m^−2^ y^−1^) than unvegetated areas and *C. nodosa* meadows and, surprisingly, comparable to *P. oceanica*. N (0.07 ± 0.01 kg m^−2^) and C_inorg_ (14.06 ± 8.02 kg m^−2^) stocks were similar between *H. stipulacea* and *C. nodosa* or unvegetated sediments, but different to *P. oceanica*. C_org_ and N stocks were higher in exotic than native *H. stipulacea* populations. Based on isotopic mixing model, organic material trapped in *H. stipulacea* sediments was mostly allochthonous (seagrass detritus 17% *vs* seston 67%). C_org_ stock was similar between monospecific and invaded *C. nodosa* meadows by *H. stipulacea*. Higher stocks were measured in the higher human pressure site. *H. stipulacea* introduction may contribute in the increase of carbon sequestration in the Eastern Mediterranean.

## Introduction

Biological invasion ranks among the most important components of global change, undermining the structure and function of native ecosystems^1^. The Mediterranean Sea receives the highest number of introduced species globally^2^ due to the opening of the Suez Canal, high maritime traffic and aquaculture, with 821 marine species being established in the region by year 2016^3^. The seagrass *Halophila stipulacea* (Forsskål) Ascherson 1867, native to the Indian Ocean and the Red Sea^4^, was first recorded in the Eastern Mediterranean Sea (Rhodes island, Greece) in 1894, following the opening of the Suez Canal in 1869, and since then it has been expanding northward and westward in the basin^5^ until Tunisia^6^, usually colonizing unvegetated sediments void of native seagrasses or macroalgae^5^, with a preliminary estimated mean expansion rate of 12 km y^−1^ ^7^. Although the ecological effect of exotic macrophytes is largely assessed^8^, the impact of *H. stipulacea* on the native ecosystems of the Mediterranean is not yet documented.

*H. stipulacea* is currently considered invasive^9^, although there is no evidence of competition with the endemic (*Posidonia oceanica* (L.) Delile) and native (*Cymodocea nodosa* (Ucria) Ascherson) seagrasses^5^, possibly due to its small shoot size compared to that of larger native species. Currently, *H. stipulacea* populations occur only in the warmer southern-eastern part of the Mediterranean basin^10^, confined by the isotherm of 15 °C, which has been considered the limit of geographical distribution of the species to the West Mediterranean^11^. However, the occurrence of *H. stipulacea* in the Mediterranean is expanding^12,13^, possibly following the increase in temperature of the region, where summer sea surface temperature (SST) has raised by 1.15 °C during the last three decades^14^. Preliminary estimations suggest that *H. stipulacea* will be present in the whole Mediterranean basin within the next 100 years^7^. Concurrently, *P. oceanica* shows mass mortality due to climatic and anthropogenic stressors^15^, whereas *C. nodosa* increases its expansion to occupy the available space derived from *P. oceanica* regression^5^, suggesting a shift in the dominant seagrass of the basin.

Carbon storage in underlying seagrass sediments is a key process in seagrass meadows. Due to intense seagrass metabolism that results in excess organic matter production^16^ part of which is accumulated in the underlying sediment^17^, high capacity to trap and retain particles^18,19^ and increased carbon preservation in sediments^20^, seagrasses bury on a global scale between 4.2 and 8.4 Pg of organic carbon in their sediments^21^, which ranks seagrass meadows among the most crucial players of Blue Carbon (BC) coastal ecosystems^22^. The capacity of seagrass ecosystems to store carbon depends on a variety of traits, such as structural complexity, primary productivity, below-ground biomass, capability to trap allochthonous matter and biogeochemical conditions of their sediments^23^. *H. stipulacea* differs in most of the aforementioned traits from the two native seagrass species of the Mediterranean, *P. oceanica* and *C. nodosa*, forming meadows of higher density but of lower below-ground biomass and production than the latter^24,25^. The simple architecture and low above- and below-ground biomass of *H. stipulacea* imply that the relative sedimentary stock of *H. stipulacea* meadows may be smaller than that of *P. oceanica* meadows, which possess a notable capacity to sequester carbon^21^. Yet, *H. stipulacea* stock may be higher than the usually smaller corresponding stock of unvegetated sediments^26,27^, due to high shoot density of the species and biomass allocation closer to the interface between water and sediment, which have been related to the high capacity of the genus *Halophila* to trap organic matter^23,28^.

Although there is a substantial focus in quantifying the capacity of several seagrass species to accumulate organic carbon and the key factors affecting storage^23,26,27,29–37^, little is known about the amount of inorganic carbon^38–40^ and even less of nitrogen^41–43^ stored in underlying sediments of different seagrass species and the factors determining their storage. Accumulation of inorganic carbon in seagrass sediments derives from sedimentation of carbonate particles^44^, calcification by associated epiphytes and fauna^45^ and active precipitation by certain seagrass species, as recently reported for *Thalassia testudinum*^46^. But the magnitude of inorganic carbon deposits depends on the balance between carbonate production and dissolution of carbonates in the canopy^47^ or sediment^48^ and environmental conditions^38^. Therefore, it is expected to differ across seagrass species and geographic regions, depending on the leaf size, leaf life span, shoot density and production and on the seawater temperature and salinity which regulate the saturation of calcium carbonate (Ω) and hence its precipitation^38^. Likewise, quantification of nitrogen stocks in seagrass ecosystems is puzzling, as nitrogen dynamics in seagrass ecosystems involves a series of biogeochemical processes between the plant, associated flora and fauna, water and sediment (uptake, nitrification, denitrification, fixation) and seagrass communities usually oscillate between being a source or sink for nitrogen^49–51^.

To our knowledge, the performance of introduced *H. stipulacea* in binding carbon and nitrogen has not been compared yet to that of native seagrass ecosystems. This is important particularly in the context of climate change, where biological invasion in combination with warming and local pressures around the coasts^14,52^ are changing the biogeography of the Mediterranean Sea^11^, which predisposes for relative changes in the magnitude of carbon and nitrogen stored in the aforementioned species across the basin. Here we examine the effect of invasion of exotic seagrass on carbon and nitrogen storage and burial in the Eastern Mediterranean Sea. We do so by comparing carbon and nitrogen storage and burial in exotic *H. stipulacea* meadows to that in endemic *P. oceanica* and native *C. nodosa* seagrass systems and adjacent unvegetated sediments at three sites in Crete island (Greece) characterized by diverse biogeochemical conditions and level of human pressure. We also compare the storage in introduced *H. stipulacea* meadows in the Mediterranean to that at two sites inside the native biogeographic range of the species (Red Sea). Lastly, we examine the isotopic composition (δ^13^C) of seagrass tissues and sediments to identify the contribution of seagrass detritus to the sedimentary organic carbon pool in the Mediterranean and Red Sea sites.

## Results

The structural (shoot density, biomass) and physiological (plant nutrient content) features differed between species and the effect was consistent among sites (Tables 1; S3). *H. stipulacea* formed denser meadows than native species at all sites. Shoot biomass showed the opposite pattern among species, with *H. stipulacea* having the lowest values and being followed by *C. nodosa*, while biomass of both was lower than that of *P. oceanica*. *H. stipulacea* had consistently higher carbon content (45.51–49.53% DW; Tables 2 and S4) than *P. oceanica* (29.36–39.12% DW) or *C. nodosa* (30.16–44.36% DW), but similar nitrogen content (0.56–1.33% DW) across the study sites. *H. stipulacea* was less ^13^C-depleted than *P. oceanica* and more than *C. nodosa*. Seagrasses leaves were enriched in nutrients at Chania, where also shoot biomass was decreased (Tables 1 and 2).

**Table 1:** Mean (±STDEV) of shoot density (shoots m^−2^) and biomass (g DW m^−2^) at each site and species.

Site	Species	Density (shoot m^−2^)	Leaf biomass (gDW m^−2^)	Rhizome biomass (gDW m^−2^)	Root biomass (gDW m^−2^)
Chania	*H. stipulacea*	11956 ± 1476Aa	8.62 ± 2.57Aa	11.42 ± 1.86Aa	7.78 ± 2.19Aa
	*P. oceanica*	247 ± 57Ab	173.57 ± 42.22Ab	115.32 ± 35.71Ab	67.43 ± 33.93Ab
	*C. nodosa*	115 ± 29Ab	5.11 ± 0.27Aa	11.72 ± 2.54Aa	7.63 ± 0.76Aa
Souda	*H. stipulacea*	3132 ± 1013Ba	4 ± 0.31Ba	4.07 ± 1.42Ba	2.66 ± 0.67Aa
	*P. oceanica*	485 ± 180Bb	325.68 ± 34.95Bb	306.33 ± 107.71Bb	86.54 ± 39.72Ab
	*C. nodosa*	491 ± 77Bb	13.61 ± 1.42Ba	41.57 ± 14.94Ba	10.68 ± 1.12Aa
Sitia	*H. stipulacea*	13148 ± 2112Aa	6.33 ± 1.56Ba	5.75 ± 1.33Ba	3.29 ± 0.95Ba
	*P. oceanica*	522 ± 96Ab	398.97 ± 49.75Bb	446.34 ± 80.61Bb	175.16 ± 8.52Bb
	*C. nodosa*	159 ± 43Ab	3.48 ± 0.17Ba	14.34 ± 3.25Ba	11.43 ± 1.06Ba

Capital and small letters indicate significant differences between sites and species, respectively (Tukey’ s post hoc test, P < 0.05. The corresponding ANOVA results are given at Table S3).

**Table 2:** Mean (±STDEV) elemental (%) and isotopic (‰) composition of carbon and nitrogen in seagrass shoots at each site and species.

Site	Species	C leaves (% DW)	C rhizomes (% DW)	C roots (% DW)	N leaves (% DW)	N rhizomes (% DW)	N roots (% DW)	δ^13^C leaves (‰)
Chania	*H. stipulacea*	48.37 ± 4.56Aa	49.09 ± 1.47a	45.19 ± 1.8a	1.28 ± 0.17A	0.46 ± 0.01Α	0.52 ± 0.04A	−9.3 ± 0.6Aa
	*P. oceanica*	39.35 ± 0.45Ab	39.25 ± 0.77b	38.74 ± 0.83b	0.88 ± 0.09A	0.62 ± 0.1Α	0.43 ± 0.04Α	−14.9 ± 0.9Bb
	*C. nodosa*	36.89 ± 0.53Ab	35.54 ± 0.04b	30.9 ± 1.32b	1.41 ± 0.02Α	0.5 ± 0.02Α	0.41 ± 0.03A	−6.5 ± 0.3Ac
Souda	*H. stipulacea*	46.78 ± 2.5Aa	46.59 ± 1.13a	43.15 ± 2.54a	0.68 ± 0.41A	1.54 ± 1.38Β	0.97 ± 0.48Β	−7.8 ± 1.2Aa
	*P. oceanica*	40.82 ± 1.42Ab	34.45 ± 12.83b	34.85 ± 14.06b	1.2 ± 0.38A	1.27 ± 0.29Β	1.21 ± 0.11Β	−13.2 ± 0.4Bb
	*C. nodosa*	40.31 ± 0.74Ab	50.46 ± 8.19b	42.31 ± 7.48b	0.88 ± 0.5Α	1.32 ± 0.72Β	1.8 ± 1.2Β	−6.4 ± 0.1Ac
Sitia	*H. stipulacea*	49.65 ± 2.05Ba	51.7 ± 1.24a	47.25 ± 1.27a	0.72 ± 0.55 Β	0.85 ± 0.65Α	0.79 ± 0.54Α	−8.4 ± 0.4Aa
	*P. oceanica*	33.74 ± 3.89Bb	28.97 ± 8.12b	25.36 ± 10.45b	0.59 ± 0.11 Β	0.81 ± 0.12Α	0.62 ± 0.16Α	−15.8 ± 1.5Bb
	*C. nodosa*	29.61 ± 2.14Bb	34.21 ± 5.68b	26.66 ± 0.85b	0.52 ± 0.3B	0.66 ± 0.43Α	0.51 ± 0.22A	−8.2 ± 0.4Ac

Capital and small letters indicate significant differences between sites and species, respectively (Tukey’ s post hoc test, P < 0.05. The corresponding ANOVA results are given at Table S4).

Granulometry analysis revealed that sediments were mainly sandy with low contribution of silt/clay (3–8%; Table S1), with the exception of South Beach (Red Sea) where 45% was gravel.

**Table 3:** ANOVA results of C_org_, C_inorg_ and N stocks between biogeographic regions, sites and habitats studied.

	Df	Mean Square	F-ratio	P- value
Two-way ANOVA (Site × Habitat) for Mediterranean Sea region				
C_org_ stock				
Site	2	0.022	9.57	0.001***
Habitat	3	0.032	13.74	<0.001***
Site × Habitat	6	0.012	5.09	0.002**
Residuals	24	0.003		
C_inorg_ stock				
Site	2	0.86	226.83	<0.001***
Habitat	3	0.03	9.20	<0.001***
Site × Habitat	6	0.07	18.38	<0.001***
Residuals	24	0.00		
N stock				
Site	2	0.003	7.82	0.002**
Habitat	3	0.003	8.31	0.001***
Site × Habitat	6	0.003	8.28	<0.001***
Residuals	24	0.0004		
One-way ANOVA (Site) for Red Sea region				
C_org_ stock				
Site	1	0.02	0.08	0.798
Residuals	4	0.24		
N stock				
Site	1	0.003	0.78	0.428
Residuals	4	0.004		
Two-way ANOVA (Biogeographic region × Site) for *H. stipulacea* habitat				
C_org_ stock				
Biogeographic region	1	43.90	113.71	<0.001***
Site	3	0.52	1.35	0.313
Residuals	10	0.39		
N stock				
Biogeographic region	1	0.30	118.41	<0.001***
Site	3	0.02	7.92	0.005**
Residuals	10	0.003		

The vertical distribution of sediment variables in core profiles was not consistent (Figs S1–S4). Mean carbon and nitrogen content in the first 20 cm of sediment differed among sites, depending on habitat (Tables S5 and S6). *H. stipulacea* sediments were enriched in C_org_ (mean content across sites 0.32 ± 0.19% DW) and N (0.03 ± 0.01% DW) compared to the unvegetated ones (C_org_ = 0.18 ± 0.05% DW, N = 0.02 ± 0.002% DW). Again, *H. stipulacea* sediment had higher C_org_ content than *C. nodosa* (0.18 ± 0.15% DW) although similar N (0.03 ± 0.01% DW), but lower than *P. oceanica* (C_org_ = 0.49 ± 0.48% DW, N = 0.09 ± 0.11% DW). C_inorg_ content was at the same range between *H. stipulacea* (5.66 ± 2.55% DW) and unvegetated sediments (5.45 ± 2.59% DW) and slightly lower than *P. oceanica* (6.57 ± 5.25% DW) and *C. nodosa* (6.47 ± 33.1% DW) sediments (Table S5). Sediments were enriched in C_org_, C_inorg_ and N at Chania (Table S5). Mean C_org_ and N content at Mediterranean *H. stipulacea* sediments were approximately 2-fold higher compared to Red Sea sites, where they averaged 0.15 ± 0.03 and 0.02 ± 0.01% DW, respectively (Table S5), without showing significant difference between sites of Eilat (Table S6). The inter-correlation of C_org_ and N sediment contents was positive in all species (*H. stipulacea* R^2^ = 0.85, P < 0.05, *P. oceanica* R^2^ = 0.87, P < 0.05 and *C. nodosa* R^2^ = 0.85, P < 0.05).

**Figure 1:**
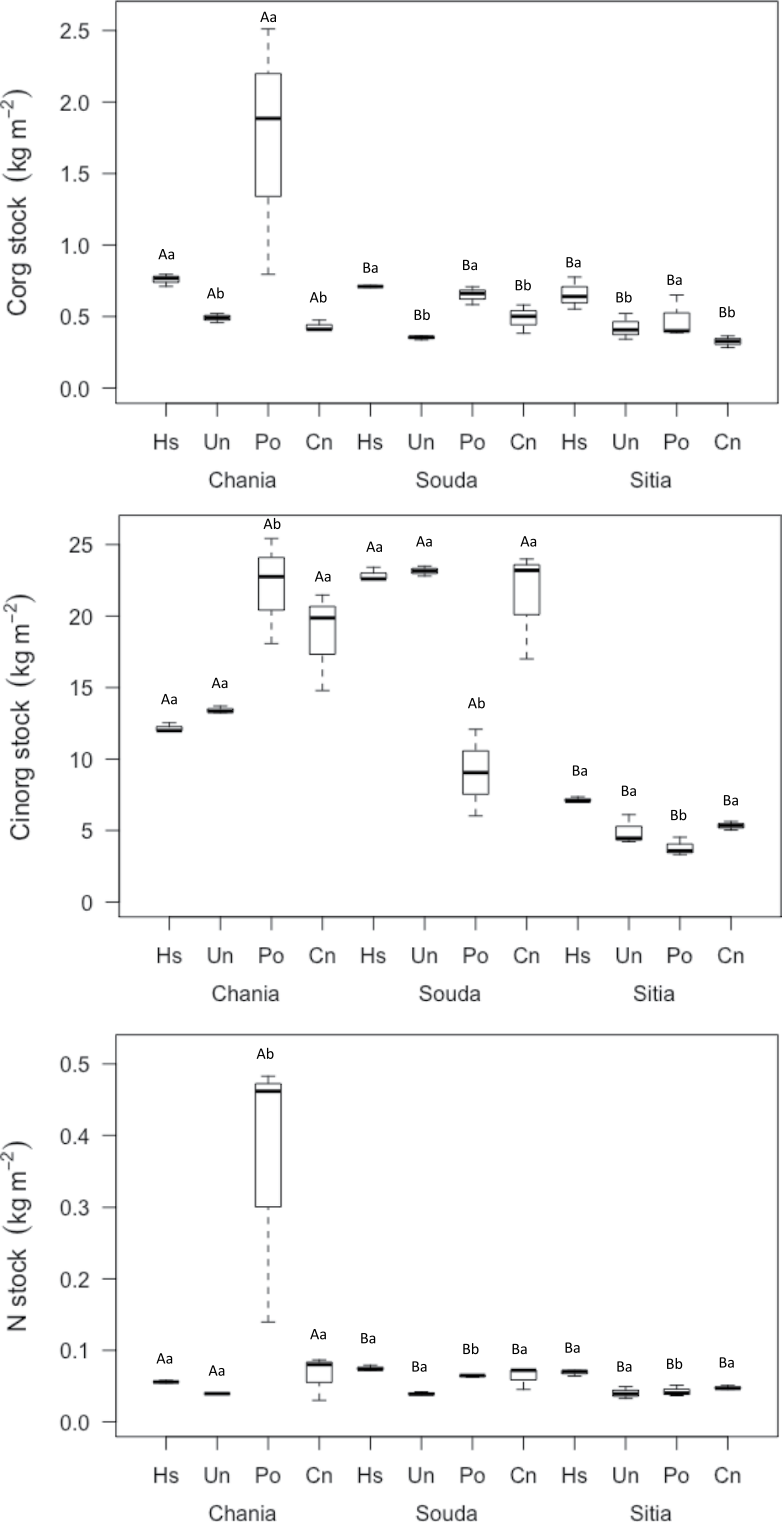
Box plots of organic and inorganic carbon and nitrogen stocks (kg m^−2^) at each habitat of the Mediterranean Sea. Significant difference (P < 0.05) between sites and habitats are given by capital and small letters, respectively.

Stocks of C_org_, C_inorg_ and N in the top 20 cm of sediment differed among habitats, but the effect depended on site (Fig. 1, Table 3). Mean C_org_ stock in *H. stipulacea* across sites (0.71 ± 0.05 kg C_org_ m^−2^ and 35 Mg C_org_ ha^−1^ in top 20 cm and top meter of sediment, respectively) was 2-fold higher than that in *C. nodosa* (0.41 ± 0.08 kg C_org_ m^−2^ and 21 Mg C_org_ ha^−1^ in top 20 cm and 1 m, respectively) and unvegetated habitats (0.42 ± 0.07 kg C_org_ m^−2^ in top 20 cm, 21 Mg C_org_ ha^−1^ in top meter) and similar (i.e. not significantly different) to *P. oceanica* (0.95 ± 0.68 kg C_org_ m^−2^ in top 20 cm, 51 Mg C_org_ ha^−1^ in top meter). Chania supporting higher stocks of C_org_ and N (Fig. 1).

*H. stipulacea* had 2-fold higher N stock (mean across sites 0.07 ± 0.01 01 kg N m^−2^ in top 20 cm, 3 Μg N ha^−1^ in top meter) than that at unvegetated habitats (mean 0.04 ± 0.01 01 kg N m^−2^ in top 20 cm, 2 Μg N ha^−1^ in top meter), similar stock to *C. nodosa* meadows (0.06 ± 0.01 kg N m^−2^ in top 20 cm, 3 Μg N ha^−1^ in top meter) and higher stock from *P. oceanica* in case of Souda and Sitia, although it was lower than the *P. oceanica* mean across sites (0.16 ± 0.18 kg N m^−2^ in top 20 cm, 8 Μg N ha^−1^ in top meter).

*H. stipulacea* supported similar (i.e. not significantly different) C_inorg_ stock (14 ± 8 kg C_inorg_ m^−2^ in top 20 cm, 709 Μg C_inorg_ ha^−1^ in top meter) to *C. nodosa* (15 ± 9 kg C_inorg_ m^−2^ in top 20 cm, 771 Μg C_inorg_ ha^−1^ in top meter) and unvegetated habitats (14 ± 9 kg C_inorg_ m^−2^ in top 20 cm, 691 Μg C_inorg_ ha^−1^ in top meter), but significantly higher stock to that of *P. oceanica* (12 ± 9 kg C_inorg_ m^−2^ in top 20 cm, 590 Μg C_inorg_ ha^−1^ in top meter).

There was no significant difference among C_org_ stock in monospecific *C. nodosa* (Chania, Souda) and *C. nodosa* meadows invaded by *H. stipulacea* (Sitia) (ANOVA, F = 4.7, P = NS).

Stocks (top 20 cm of sediment) of *H. stipulacea* were similar within Red Sea sites (Table 3), with 0.36 ± 0.03 kg C_org_ m^−2^ and 0.04 ± 0.008 kg N m^−2^ at North Beach and 0.35 ± 0.06 kg C_org_ m^−2^ and 0.04 ± 0.006 kg N m^−2^ at South Beach.

**Figure 2:**
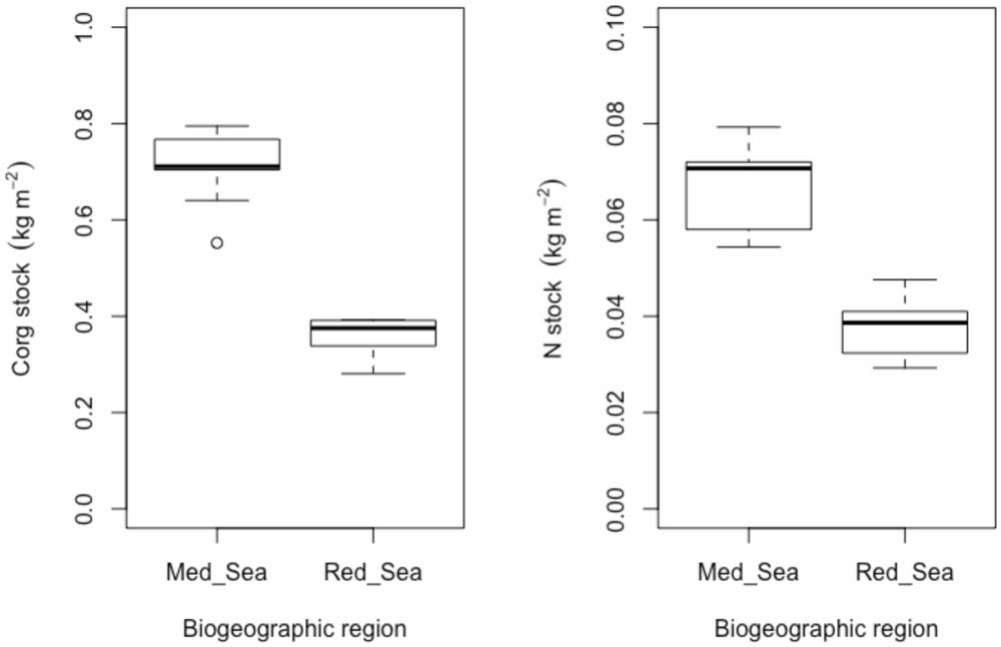
Box plots of organic carbon and nitrogen stocks (kg m^−2^) in *H. stipulacea* meadows of the two biogeographic regions.

Stocks of C_org_ and N in *H. stipulacea* sediments differed between biogeographic regions (Table 3; Tukey’s post hoc test Chania ≠ Souda) by almost 2-fold. They ranged between 0.55 and 0.79 kg C_org_ m^−2^ with a mean of 0.71 kg C_org_ m^−2^ and 0.05 and 0.08 kg N m^−2^ with a mean of 0.07 kg N m^−2^ at Mediterranean Sea sites and between 0.28 and 0.39 kg C_org_ m^−2^ with a mean of 0.36 kg C_org_ m^−2^ and 0.03 and 0.05 kg N m^−2^ with a mean of 0.04 kg N m^−2^ at Red Sea sites (Fig. 2).

**Figure 3:**
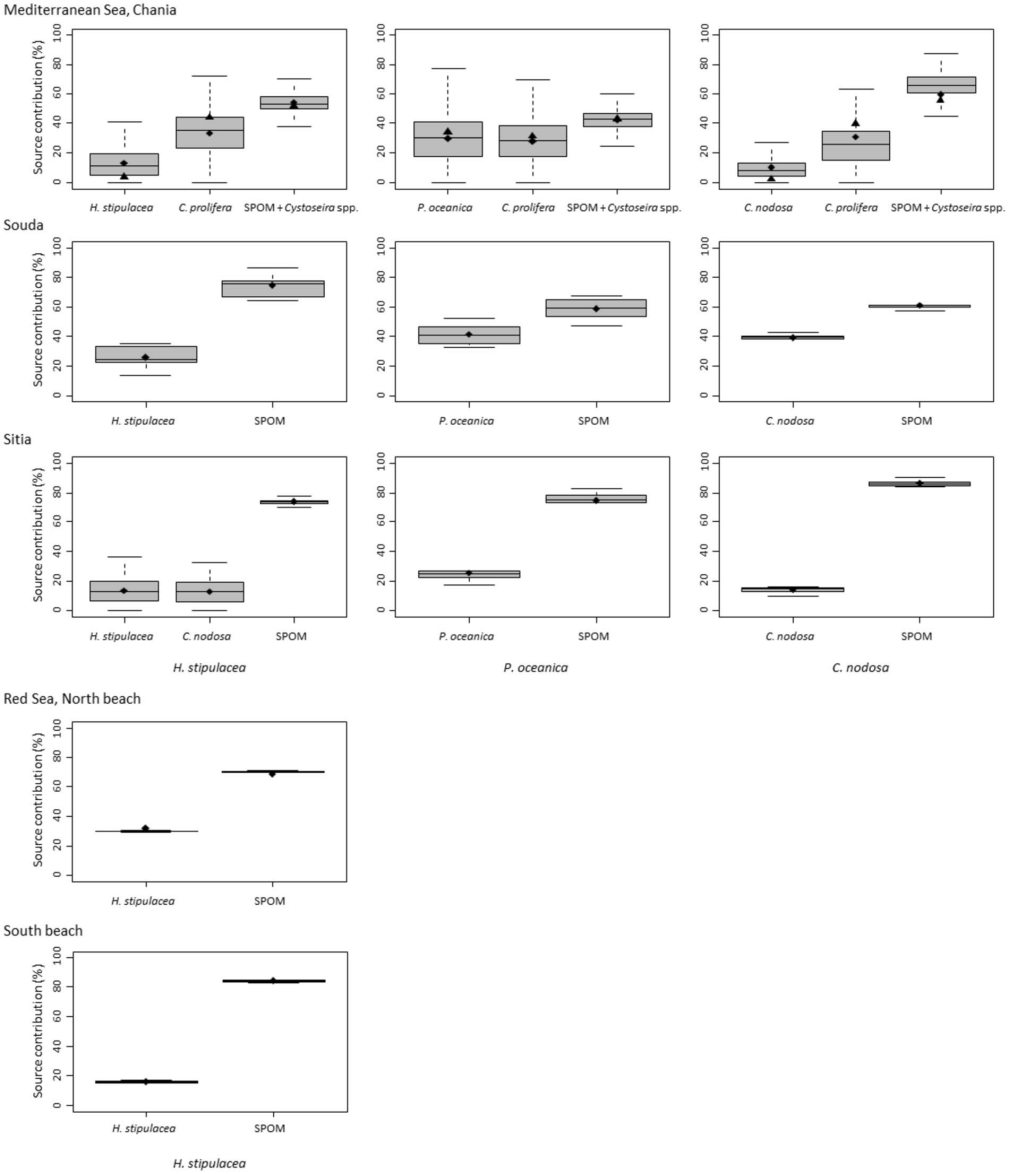
Percentage contribution of end-members (grouped when their δ^13^C was not significantly different, see Materials and methods section) to the first 5 cm of sediment organic carbon of each habitat and site investigated. Each box contains 50% of the data, the thick horizontal line indicates the median; lower and upper whiskers of the boxes represent respectively the lowest and the highest values of the total range of the distribution. Black diamonds show the mean value, black triangles show the mode, where available.

According to the mixing model, most of the sedimentary C_org_ in the meadows examined derived from allochthonous sources (i.e. SPOM and/or *Cystoseira* sp.), with their contribution ranging from 42% at Chania to 86% at Sitia (mean = 66%) in the Mediterranean meadows and from 70% at North Beach to 84% in South Beach (mean = 77%) in the Red Sea meadows (Fig. 3). *C. prolifera* present only at Chania sites contributed with 29% in mean. Among seagrass species, *H. stipulacea* showed the lowest contribution to sedimentary C_org_, ranging from 13% at Chania and Sitia to 26% at Souda (mean of 17%), compared to *C. nodosa*, that contributed 9%, 39% and 14% at Chania, Souda and Sitia, respectively (mean of 21%) and *P. oceanica*, that showed the highest contribution, contributing 30%, 41% and 25% at Chania, Souda and Sitia sedimentary stocks, respectively (mean 32%). *H. stipulacea* detritus contributed by 30% and 16% at North and South Beach, respectively, with a mean of 23% at Red Sea sites. Posterior distributions of possible solutions for all end-members used in each Bayesian mixing model (i.e. for the three habitats of Chania and Sitia in presence of *H. stipulacea*) are reported in Fig. S6.

**Figure 4:**
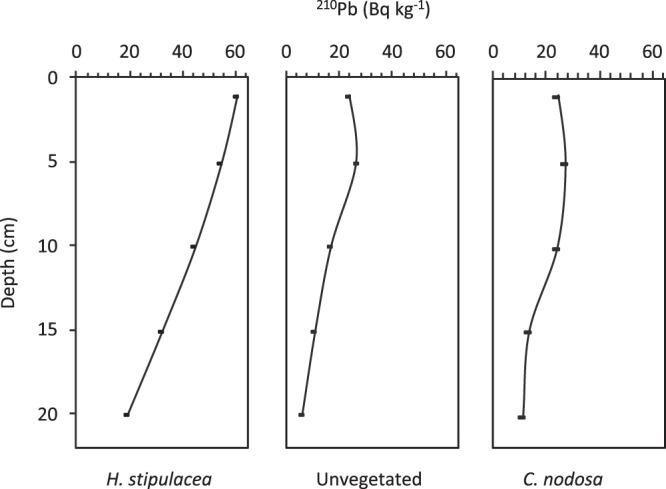
^210^Pb activity (Bq kg^−1^) with sediment depth for *H. stipulacea*, unvegetated and *C. nodosa* habitats at Chania (only significant activities are shown).

Based on the ^210^Pb activities (Fig. 4), sediment accumulation rate at Chania was 0.39 cm y^−1^ in *H. stipulacea* and 0.3 cm y^−1^ in *C. nodosa* meadows and 0.2 cm y^−1^ in unvegetated sediment. The derived age of the 20 cm sediment depth corresponed to approximately 51 y in *H. stipulacea*, 100 y in unvegetated and 67 y in *C. nodosa* meadow. The burial rate of C_org_, C_inorg_ and N was 2–3 times higher at *H. stipulacea* meadow (14.78 gC_org_ m^−2^ y^−1^, 237 gC_inorg_ m^−2^ y^−1^ and 1.09 gN m^−2^ y^−1^) compared to those at the unvegetated sediment (4.91 gC_org_ m^−2^ y^−1^, 134 gC_inorg_ m^−2^ y^−1^ and 0.39 gN m^−2^ y^−1^). The C_org_ burial rate of *H. stipulacea* was again 2-fold higher but C_inorg_ and N burial rates were similar to that of *C. nodosa* (6.46 gC_org_ m^−2^ y^−1^, 281 gC_inorg_ m^−2^ y^−1^ and 0.99 gN m^−2^ y^−1^).

## Discussion

The exotic *H. stipulacea* meadows studied here supported notable sedimentary C_org_ stocks compared to native seagrass, as well as to adjacent unvegetated sediments. Higher stocks in seagrass compared to bare sediments has been reported elsewhere, with 3 to 11-fold higher C_org_ stocks of *P. sinuosa* meadows in Australia^27^ and 2 to 4-fold higher stocks of *Thalassia testudinum* and *Halodule wrightii* meadows in Gulf of Mexico^26^ than the corresponding bare sediments. Mean C_org_ content in *H. stipulacea* sediments was similar to values reported from Greece before (0.37 ± 0.3% DW^25^). *C. nodosa* sediments were particularly poor in sedimentary C_org_ with a mean content similar to estimates from Cadiz Bay (Spain) (0.15% DW^17^ Supplement Material) and Greece (0.25 ± 0.3% DW^25^), which resulted in lower sedimentary stock. There are no available estimates of *C. nodosa* stocks around Mediterranean, but further studies are necessary given the current progression of species distribution^5^ and also the potential shift in its distribution following change in thermal conditions of the basin^53^. Mean C_org_ in *P. oceanica* sediments were on the same range of that from Greek waters before (0.4 ± 0.19% DW^54^ and 0.35 ± 0.5% DW^25^). C_org_ was comparable to data from Italy (3.09 ± 2.2% DW, eastern Sicily; S. Vizzini, unpubl. data), but still ranged lower than the mean from Spain (3.91% DW, N = 217^55^), which possibly represents the most complete compilation of relative data, suggesting large variability in C_org_ content in *P. oceanica* sediments, which is reflected in the high variability of stocks calculated for the species across Mediterranean. The mean (across sites) sedimentary stock of *P. oceanica* here was lower than the mean (167 ± 65 Mg C_org_ ha^−1^ in the top meter^34^) from previous measurements obtained in Western Mediterranean meadows (Balearic Islands) and even less than that in *P. oceanica* reefs (372 ± 65 Mg C_org_ ha^−1^ ^21^) from the same region, where the plant grows vertically for many meters down into the sediment^56^. Yet, the surprisingly comparable sedimentary C_org_ stock of *H. stipulacea* to that of *P. oceanica* meadows suggest considerable capacity of the former species to accumulate carbon in relation to its native counterpart.

In accordance with ours results, the stocks of meadows formed by the congeneric species of those of our study, *H. ovalis* and *P. australis* in Australia, did not differ despite the big difference in their shoot size, and *H. ovalis* stock was the second highest among 10 seagrass species studied there^23^. This result was attributed to the high capacity of *Halophila* to trap organic matter, but also to its preference to naturally inhabit depositional environments. This is consistent with the results found in *H. stipulacea* meadows examined in this study, where sediments were mainly composed of seston and less of seagrass detritus (67% *vs* 17% contribution to organic matter pool, respectively), suggesting that high carbon sequestration capacity of the species depends more on the deposition and trapping of allochthonous material^17^ and less on its below-ground biomass and productivity, which are at the lower end range provided for seagrass species^24^.

The high capacity of *H. stipulacea* to trap allochthonous matter possibly relates to its high density and small size*. Halophila* sp. reach high densities and *H. stipulacea* in particular forms far denser meadows (12,795–25,345 shoots m^−2^ ^57^; 10,500 ± 2,700 shoots m^−2^ ^12^; 476–9,900 shoots m^−2^ ^6^, 1,506–6,092 shoots m^−2^ ^25^, this study) than *P. oceanica* or *C. nodosa* (e.g. 244–700 shoots m^−2^ and 544–2,331 shoots m^−2^, respectively^25^, this study). In addition, the smaller leaves of *H. stipulacea* than other seagrass species (Leaf Area Index = 0.82, 0.95 and 6.39 m^2^ leaves m^−2^ for *H. stipulacea*, *C. nodosa* and *P. oceanica*, respectively^25^) and the relatively simple architecture of *H. stipulacea*^58^ with leaf and rhizome biomass allocation closer to interface between sediment and water may result in significant sediment stabilization effects, as shown for the congeneric species *H. decipiens*^28^. An experimental study showed that *Caulerpa* sp. beds, an alga of small size similar to *H. stipulacea*, had equal or even higher capacity to trap particles than *P. oceanica* or *C. nodosa* beds, as small size and high density of *Caulerpa* sp. shoots induced a ‘skimming flow’ over the beds and increased transport of particles to the canopy^59^. In our case, the 2 to 3-fold higher burial rate of *H. stipulacea* meadow compared to that of unvegetated and *C. nodosa* habitats, a rate that actually falls in the reported range for the *P. oceanica* meadows of Balearic Islands, Western Mediterranean (9 and 52 gC_org_ m^−2^ y^−1^ ^34^), indicates a considerable capacity of *H. stipulacea* to trap particles. Nevertheless, as estimates of burial rate in seagrass meadows are still limited^26,27,34,60^ (despite the 4-fold rapid increase in publication effort regarding seagrass carbon storage^61^) and our estimate derives from one site, the burial rate given here should be considered a preliminary estimate of the capacity of *H. stipulacea* to bury carbon.

The capacity of *H. stipulacea*, but also of the native species, to store N was substantial. To our knowledge, despite the increase in C_org_ stock data, there are only a couple of estimates of N stock in any seagrass species. A mean estimate of 12.4 ± 1.1 Mg N ha^−1^ has been provided, encompassing values from *Thalassia testudinum* meadows across Florida Bay and *Amphibolis antarctica* and *Halodule uninervis* meadows from Shark Bay^41^. A modeled seagrass recovery following a large restoration effort in coastal bays of Virginia yielded 170 ton N yr^−1^ via denitrification^42^, suggesting a significant removal of N from the ecosystem by the holobiont. Despite the parallel accumulation of organic carbon and nitrogen in sediments, the trend of C_org_ and N stocks between habitats differed, suggesting species specific differences. Content of N in *H. stipulacea* and *C. nodosa* sediments were at a similar range with values from the Eastern Mediterranean before (0.04 ± 0.03% DW and 0.02 ± 0.03% DW, respectively^25^), but sediment N in *P. oceanica* was higher than previously reported (0.03 ± 0.01% DW^54^ and 0.04 ± 0.01% DW^25^), resulting in significant higher N stock in the latter species. Very recently, the quantification of N stock of several *Z. marina* meadows across Denmark showed that this stock is highly variable in space (24–448 g N m^−2^), depending on sediment characteristics (i.e. grain size) and nutrient availability^43^. Complex N dynamics in seagrass ecosystems, namely N mineralization and fixation^49–51^, along with high variability of N in tissues of different seagrass species^62^, which are both affected by species specific differences and environmental conditions^25,63^, could result in highly variable N content in underlying sediments^17^ and thus diverse stocks across species and regions, but further studies are needed to elucidate this.

The higher amount of C_org_ but similar C_inorg_ stored between *H. stipulacea* and unvegetated habitats suggest that the accumulated C_inorg_ does not derive solely from calcification inside the meadow. Environmental factors such as temperature and salinity favor carbonate precipitation by other benthic organisms inhabiting or visiting  the meadow^38^. C_inorg_ stock of *H. stipulacea* meadows fell very close to the global mean for seagrasses (654 ± 24 Μg C_inorg_ ha^−1^ ^38^), but it was far higher than the mean value calculated for *Halophila* genus so far (304 Μg C_inorg_ ha^−1^), expanding the range of C_inorg_ stock for the genus. Despite the smaller leaf size and life span^24,25^ and lower load of calcareous epiphytes in *H. stipulacea* meadows, the C_inorg_ stock was higher compared to that in *P. oceanica*, suggesting that shoot density more than shoot size affected the storing capacity here. In fact, accumulation of C_inorg_ was considerable also in *C. nodosa* compared to *P. oceanica*, both supporting similar stocks given for the corresponding genus (738 Μg C_inorg_ ha^−1^ and 563 Μg C_inorg_ ha^−1^, respectively^38^). A global review of C_inorg_ stocks in seagrass ecosystems did not find a clear effect of genera size on the amount of C_inorg_ stored, with some small genera supporting large stocks and *Posidonia* in particular supporting intermediate stocks^38^.

The higher C_org_ and N stocks of *H. stipulacea* meadows across sites, but also of both native species, were measured at Chania, where the meadows receive the highest human pressure among the studied sites, as shown by the highest Pressure Index measured at this site^64^ and the mesotrophic conditions. An increase in C_org_ burial was also measured at *P. oceanica* meadows of Mallorca (Spain) since the onset of anthropogenic pressure and particularly at sheltered areas with high human activity^34^, which was related to the increase in contribution of seston to the organic pool of seagrass sediments as a result of general eutrophication associated with the intensification of coastal anthropic activities^65^. Here we did not measure any increase in the SPOM contribution at Chania, but we did observe rich macroalgal communities (*Caulerpa prolifera*), which contributed, on average, by 29% to the sediment organic pool, and mesotrophic conditions, as shown by the relatively high Chl*a* concentration in the water column, suggesting relatively nutrient enriched conditions at the specific site. This was also consistent with higher sediment C_org_ and nutrient content measured in seagrass tissue at Chania, which is indicative of increased nutrient availability to seagrass meadows^66^.

The lack of significant difference among C_org_ stocks in monospecific *C. nodosa* and invaded meadows by *H. stipulacea* suggests that the invasion of *H. stipulacea* did not affect negatively the carbon stock of the natives. Nevertheless, a progression in its distribution could be expected as a result of warming^67^, which may trigger changes in the balance between the exotic seagrass and its native counterparts. Despite the lack of evidence of invasiveness of *H. stipulacea* in the Mediterranean^5,68^, expansion of the species to the Caribbean^69^ had detrimental effect on native seagrass abundance in certain occasions. Manipulation experiments showed that *H. stipulacea* expanded rapidly between transplanted shoots of native *Syringodium filiforme* in the Caribbean, which resulted in replacement of the native species^70^. In addition, *in situ* measurements showed that the native *Thalassia testudinum* was negatively affected by *H. stipulacea*, when the latter reached high densities as a result of nutrient enrichment of the area^71^. Therefore, potential overexpansion of *H. stipulacea* with a parallel regression of *P. oceanica* as a result of warming^15,72^ would provide a competitive advantage to *H. stipulacea* over the native seagrass or even lead to replacement of the latter that would result in a substantial reduction in type and amount of ecosystem services provided by seagrass in the region^73^, including considerable carbon storage by the endemic *P. oceanica*^61,74^. Hence, although the introduction of *H. stipulacea* does not seem catastrophic at the moment, we need to shift our attention from focusing on the properties of the invading organism to how anthropogenic and climate change impacts on native ecosystems may facilitate the invasion^1^.

*H. stipulacea* formed denser but lower biomass meadows in the Mediterranean compared to observations from the Red Sea, indicating that species performance may vary in and outside its natural biogeographic range. Shoot density in the Mediterranean sites (9,412 shoots m^−2^) was higher from values previously reported from Greece (3,499 shoot m^−2^ ^25^) or Red Sea (3,198 shoots m^−2^ ^75^ and 1,568 shoots m^−2^ ^76^ from the Jordanian coast of Gulf of Aqaba at similar depth range), whereas biomass was far lower^29,76^. However, the 2-fold higher C_org_ and N stocks of Mediterranean *H. stipulacea* meadows compared to Red Sea ones were not related to the difference in living plant biomass [0.08 Mg C ha^−1^ in Mediterranean sites (this study) and 0.51 Mg C ha^−1^ Red Sea sites (M.C. Gambi, G. Winters, S. Vizzini, unpubl. data)], as no such correlation between living C and sediment C_org_ stock was found (data not shown). Similar results with C_org_ stocks being independent of living C stock were given for *H. stipulacea* meadows of Arabian Gulf^29^ and for other seagrass species^39^. The higher stocks were possibly related to specific local geomorphological and hydrological conditions, which resulted in almost double mean C_org_ contents in Mediterranean sediments compared to Red Sea ones, although both values fell inside the range reported at similar depths from the natural biogeographic distribution of the species (0.26–0.60% DW^77^ and 0.10–0.45% DW^78^ from Gulf of Aqaba, Israel; <0.05–2.44% DW along the Abu Dhabi coasts, United Arab Emirates^29^). Stocks of Red Sea *H. stipulacea* meadows studied here (17 Mg C_org_ ha^−1^ at the top meter) are lower than reported before (58–92 Mg C_org_ ha^−1^ at the top meter of mixed meadows in United Arab Emirates with 20–63% cover of *H. stipulacea*^29^ and 31 Mg C_org_ ha^−1^ at the top meter in Saudi Arabia^79^), possibly due to low seagrass productivity and the high energy environment of the sites studied here, as seen by the mainly coarse grain size. A positive inter-relation between mud content and soil C_org_ is found in sediments where the contribution of seagrass-derived C_org_ to the sedimentary pool is relatively low, such as in small and fast-growing meadows formed by small species like *Halophila* sp.^55^.

Our findings show that *H. stipulacea*, as well as *P. oceanica* and *C. nodosa* meadows support higher stocks than unvegetated sediments, contributing in the offset of carbon emissions and helping in mitigation of climate change in the region. Most importantly, *H. stipulacea* meadows expanding at the Mediterranean sites could possess comparable or even higher storing capacity compared to the native counterparts, suggesting that introduction of *H. stipulacea* potentially contributes in increase of carbon sequestration in the Eastern Mediterranean. However, the fact that organic carbon deposited in *H. stipulacea* sediments is mainly allochthonous renders this introduced carbon stock more susceptible to remineralization and especially when compared to *P. oceanica* stocks, as SPOM is more labile than seagrass tissue^65,80^, implying a deterioration in the quality and quantity of carbon ultimately buried in the region. Furthermore, the weak rhizome structure of the species, particularly as opposed to that of *P. oceanica*, suggests enhanced probability of sediment erosion and subsequent loss of sedimentary C_org_ stock. Lastly, a better understanding of the effect of this exotic species on the native biodiversity, and, importantly, how this effect may change in the context of future warming of the region are a prerequisite, before we can account the full size of *H. stipulacea* sedimentary stock in the Mediterranean Sea.

## Materials

### Sampling strategy

The study was conducted at Crete Island, Greece (Mediterranean Sea) and Eilat Bay, Israel (Red Sea) (Table 4). We selected three sites in Crete (Chania, Sitia and Souda), where all seagrass species formed monospecific stands (except Sitia, where *H. stipulacea* expanded inside some *C. nodosa* patches), and two sites at Eilat (North Beach and South Beach), where only *H. stipulacea* was present. We sampled all seagrass species and adjacent unvegetated sediments at each site in Crete and only *H. stipulacea* in Eilat. Seagrasses were the only macrophytes present at the sites, with the exception of Chania, where *Caulerpa prolifera* and *Cystoseira* spp. were found adjacent to the seagrasses. The sites were visited during the warm season, when temperature range was 20–26 °C. The depth range was 5–21 m, well above the lower depth limit of the seagrass (90 m^81^), and sediments were mainly sandy.

The sites were characterized by different level of human pressure. Chania is under moderate human pressure by cumulative impacts (i.e. sewage discharge, agriculture run-off, industrial/chemical pollution, eutrophication and harbor/marina/ports) with a Pressure Index (which quantifies all the pressures exerted in the water bodies) of 0.78^64^. Souda is also affected by similar pressures (sewage, agriculture, industry, maritime traffic) but to a lower extent (Pressure Index = 0.56^64^). Pressure Index is not available for Sitia, but the site should be considered unaffected, situated in a non-urbanized bay with no coastal activity. North Beach is under high human pressure, namely extended coastal infrastructures and highly populated beaches, while South Beach is relatively unaffected^77^. The annual mean chlorophyll *a* (Chl*a*) concentration of the water column (satellite data; Oceancolor web Aqua MODIS L3-SMI 4 km) during the corresponding sampling years (2013 and 2014 for Mediterranean and Red Sea sites, respectively) for Chania, Souda, and Sitia and for North and South Beach was 0.12 μg l^−1^, 0.15 μg l^−1^, 0.09 μg l^−1^, 0.17 μg l^−1^ and 0.16 μg l^−1^, respectively, classifying the particular sites as lower mesotrophic (Chl*a* = 0.1–0.4 μg l^−1^), except Sitia which was classified as oligotrophic (Chl*a* < 0.1 μg l^−1^)^82^.

Shoot number of *P. oceanica* and *C. nodosa* was measured *in situ* by divers at each site from five randomly thrown 40 cm × 40 cm quadrates. Divers also collected *P. oceanica* and *C. nodosa* shoots by hand (3 replicates, 10 shoots per replicate), to measure biomass at the laboratory. Shoot density and biomass of *H. stipulacea* were estimated at the laboratory from shoots collected by divers using an aluminum core (5 replicates, i.d. 15 cm).

Sediment cores (3 replicates, i.d. 4.5 cm D each) were collected from each site down to 20 cm sediment depth, to allow the reconstruction of recent sediment records. Sediment was sliced in various depth intervals (0–1, 1–5, 5–10, 10–15 and 15–20 cm). Sediment compaction, which is inevitable when coring^30^, was measured as the difference between the outer and the inner distance from the top of the core to the sediment surface divided by the core length inserted in the sediment and was very small (3–6%).

**Table 4:** Sampling design and site characteristics [depth (m) and grain size (% sand − % silt/clay)].

Biogeographic region	Site	Habitat	Time	Depth
Mediterranean Sea	Chania (35°33′55″N 24° 4′48″E)	*H. stipulacea*	June 2013	16 m
		Unvegetated		16 m
		*P. oceanica*		21 m
		*C. nodosa*		16 m
	Souda (35°28′17″N 24° 8′54″E)	*H. stipulacea*	June 2013	5 m
		Unvegetated		5 m
		*P. oceanica*		5 m
		*C. nodosa*		5 m
	Sitia (35°12′26″N 26° 0′18″E)	*H. stipulacea*	Sept 2013	10 m
		Unvegetated		10 m
		*P. oceanica*		10 m
		*C. nodosa*		10 m
Red Sea	North Beach (29°32′46″N 34°57′53″E)	*H. stipulacea*	June 2014	9 m
	South Beach (29°29′51″N 34°54′45″E)	*H. stipulacea*	June 2014	9 m

*Data from^77^.

### Laboratory analysis

The seagrass shoots were transferred to the laboratory where the leaves were gently scraped with a razor blade to remove epiphytes, and seagrass modules (leaves, rhizomes, roots) were dried at 60 °C for 48 h. Dried tissue was acidified (HCl, 2 N) to remove carbonates. Carbon and nitrogen content and δ^13^C were analyzed in an Elemental Analyzer (Thermo Scientific Flash EA 1112) connected to an Isotope Ratio Mass Spectrometer (Thermo Scientific Delta Plus XP) and expressed in % and δ unit notation (‰ deviations from the international standard Vienna Pee Dee Belemnite), respectively.

Sediment from each slice was dried at 60 °C for 48 h and ground. Grain size analysis was performed using wet sieving to separate the sand fraction (>63 μm), whereas the finer silt and clay fractions (63 to 0.1 μm) were analyzed with a Sedigraph (Micromeritics 5100).

Dried sediment samples were weighed in silver (for organic carbon, C_org_, analysis) and tin (for total carbon C_tot_ and total nitrogen N analysis) capsules and analyzed as above. C_tot_ and N were analyzed on sediment as it is, while C_org_ was analyzed on sediment acidified with HCl (18%) added drop by drop to remove carbonates. Inorganic carbon (C_inorg_) was estimated as the difference between C_tot_ and C_org_. δ^13^C was analyzed in acidified (HCl, 2 N) sediment. The analytical precision of the δ^13^C measurement based on the standard deviation of replicates of the internal standard δ^13^C (International Atomic Energy Agency IAEA-CH-6) was 0.1‰.

### Calculations

Shoot density (shoots m^−2^) was estimated as the number of shoots divided by the sampled area for each species.

Biomass (g DW m^−2^) of each seagrass module was estimated as the product of dry weight per shoot and shoot density.

Dry bulk density (g cm^−3^) was calculated as the dry weight of sediment divided by the volume of the wet sample.

Stock of organic and inorganic carbon and nitrogen (g cm^−2^) at the top 20 cm of sediment was estimated as:

$${\rm{Stock}}={\rm{\Sigma }}({C}_{i}\times bi\times di),$$

where C*i* is the concentration of C_org_ or C_inorg_ or N (in % DW divided by 100), b*i* is the dry bulk density (in g cm^−3^) and d*i* is the sediment depth (in cm) of the sediment slice *i*. The stocks were converted to kg m^−2^ and Mg ha^−1^ to compare with similar studies.

For the calculation of the sediment accumulation rates, the down core total ^210^Pb activity was determined through the activity of its alpha-emitting granddaughter ^210^Po, assuming secular equilibrium with ^210^Pb. For the total dissolution of the dried sediments the analytical method described by^83^ was applied. The supported ^210^Pb activity was determined through the activity of its parent ^226^Ra (assuming they are in equilibrium), which was measured in a High Purity Germanium detector (HPGe) with nominal relative efficiency 50% (ORTEC GEM-FX8530P4). The ^210^Pb_xs_ activity was calculated from the difference between the total ^210^Pb activity and that of the background. For the calculation of the rates the Constant Flux Constant Sedimentation model (CFCS)^84^ was used. The down core activities of ^210^Po were measured in all the study sites, however, in most cases it was not feasible to calculate any accumulation rate as the ^210^Pb activities were too low or the sediment cores were bioturbated. The supported ^210^Pb activity was determined only in the case of Chania (*H. stipulacea, C. nodosa* and unvegetated), in order to calculate the relative rate. Due to the coarse character of the sediments and the short length of the cores, it was not possible to use ^137^Cs as an independent tracer.

### Data analysis

The Shapiro-Wilk test was used to check if the data were normally distributed. Cochran’s test was used in order to check the heterogeneity of variance in seagrass and sediment variables prior to performing Analysis of Variance (ANOVA). Data were log-transformed when necessary. A two-way ANOVA was used to detect possible statistical differences between sites and habitats or biogeographic regions^85^. In case of significant difference between levels (P* < *0.05), a Tukey’s post-hoc test was used to show which level differed. Regression analysis was used in order to detect a trend in the distribution of sediment properties with depth. The aforementioned analyses were performed using R version 3.3.3 (R Core Team, 2017).

Mixing models were used to estimate the contribution of potential sources of organic matter to the carbon isotopic composition (δ^13^C) of sediment, hence to elucidate the origin of C_org_ present. Mixing models were restricted to the first 5 cm of sediment (hence using data available for 0–1 and 1–5 cm layers); deeper and older layers were excluded because they may have undergone isotopic alterations during post-depositional decomposition of seagrass tissues, which instead are considered null or negligible in surficial layers (e.g.^39,86^). Models were run separately for each site and habitat. Only end-members present at each site and habitat were included in the models as observed during sample collection. These were: different species of seagrasses, suspended particulate organic matter (SPOM) as a proxy for phytoplankton, and macroalgae (*Caulerpa prolifera* and *Cystoseira* spp. present only at Chania habitats). Seagrass δ^13^C data were analyzed in the present study, with the exception of those of *H. stipulacea* from the Red Sea sites, which were provided by M.C. Gambi, G. Winters, S. Vizzini (unpubl. data, Table S2). The δ^13^C signature of SPOM was obtained from the literature, by averaging values of Mediterranean coastal areas^87,88^ (Table S2). Macroalgae δ^13^C data, specifically for *Caulerpa prolifera* and *Cystoseira* spp. were obtained respectively from S. Vizzini (unpubl. data) and^87^ (Table S2). When the number of end-members considered was two (one seagrass species and SPOM), a two-source mixing model was adopted based on the following equation by^89^: contribution of source 1 (%) = (δ^13^C_sediment_ − δ^13^C_source 2_) × 100/(δ^13^C_source 1_ − δ^13^C_source 2_). This was the case of all the three habitats of Souda, two out of three habitats of Sitia (*P. oceanica* and *C. nodosa* habitats) and both the Red Sea sites. When the number of end-members considered was higher than two, Bayesian mixing models were used (R package SIAR: Stable Isotopes Analysis in R^90^). This was the case of the three habitats of Chania and the *H. stipulacea* habitat of Sitia. Differences in δ^13^C among end-members were tested through Permutational Analysis of Variance (PERMANOVA) based on the Euclidean distance matrix and when they were non-significant (P > 0.05) end-members were grouped according to^91^ to reduce sources of uncertainty that could influence mixing model results when using multiple end-members. The only end-members grouped were SPOM and *Cystoseira* spp. at the three habitats of Chania.

## Supplementary information

Dataset 1

## Data Availability

Data supporting this study will be available upon request to EA.
