# Supplementary material for: Exotic Halophila stipulacea is an introduced carbon sink for the Eastern Mediterranean Sea
Source: Sci Rep. 2019 Jul 3;9:9643. doi: 10.1038/s41598-019-45046-w (PMC6610076; doi:10.1038/s41598-019-45046-w)
Supplement: Supplementary file 1 — Dataset 1 [file 41598_2019_45046_MOESM1_ESM.pdf]

Supplement material

Exotic *Halophila stipulacea* is an introduced carbon sink for the Eastern Mediterranean Sea

Eugenia T Apostolaki, Salvatrice Vizzini, Veronica Santinelli, Helen Kaberi, Cristina Andolina, Evangelos Papathanassiou

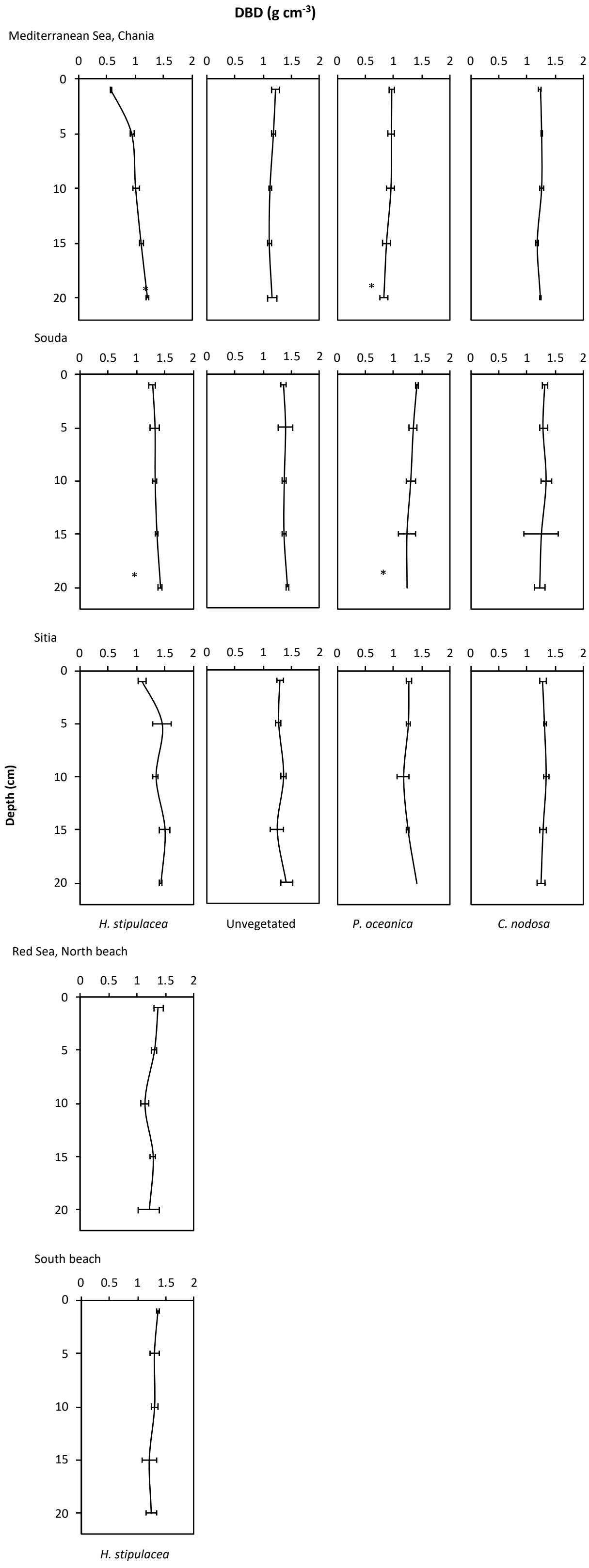

Figure S1. Vertical profile of dry bulk density (DBD) in sediment at each habitat and site. Asterisks indicate significant linear trends with sediment depth.

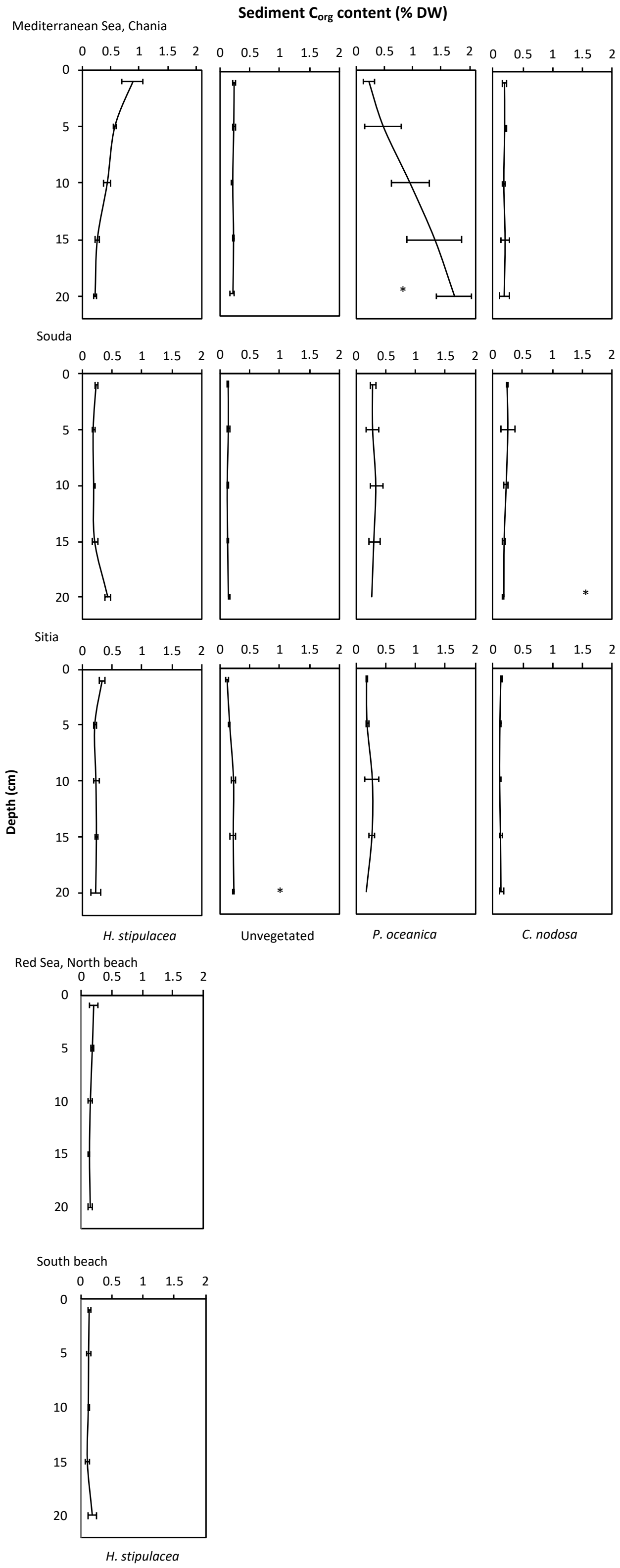

Figure S2. Vertical profile of organic carbon content (C_{org}) in sediment at each habitat and site. Asterisks indicate significant linear trends with sediment depth.

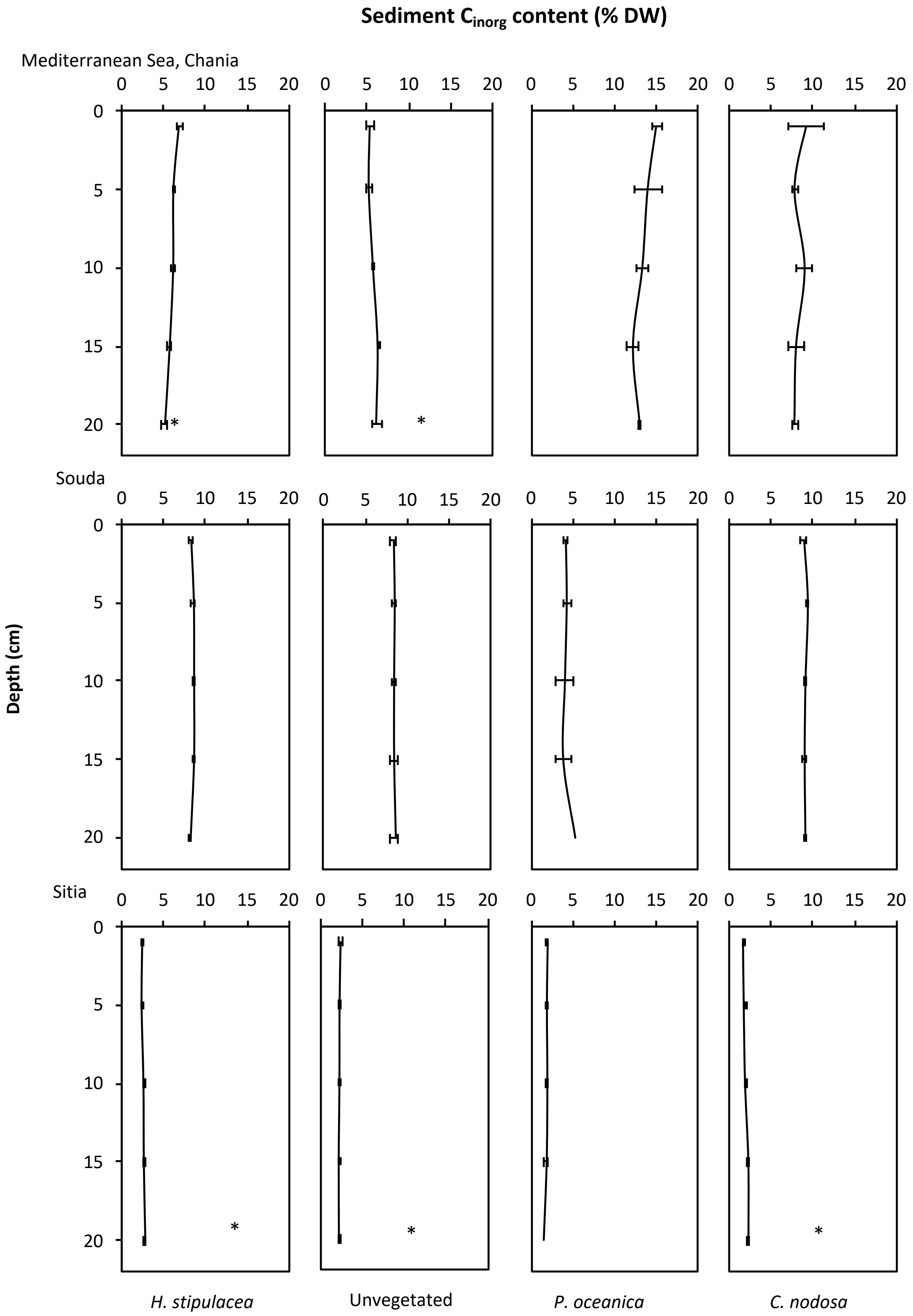

Figure S3. Vertical profile of inorganic carbon content (C_{inorg}) in sediment at each habitat and site. Asterisks indicate significant linear trends with sediment depth.

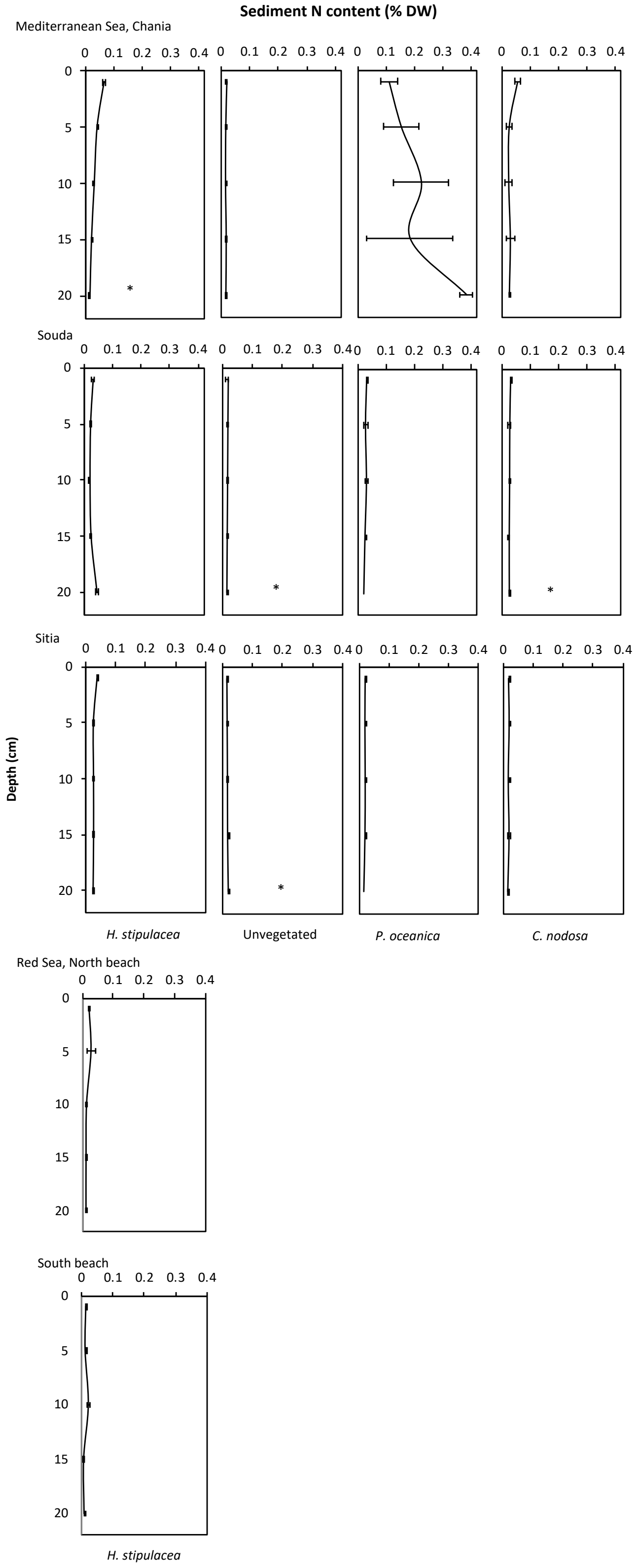

Sediment N content (% DW)
Mediterranean Sea, Chania
Souda
Sitia
Depth (cm)
0
5
10
15
20
0 0.1 0.2 0.3 0.4
*
H. stipulacea
Unvegetated
P. oceanica
C. nodosa
Red Sea, North beach
South beach
H. stipulacea

Figure S4. Vertical profile of nitrogen content (N) in sediment at each habitat and site. Asterisks indicate significant linear trends with sediment depth.

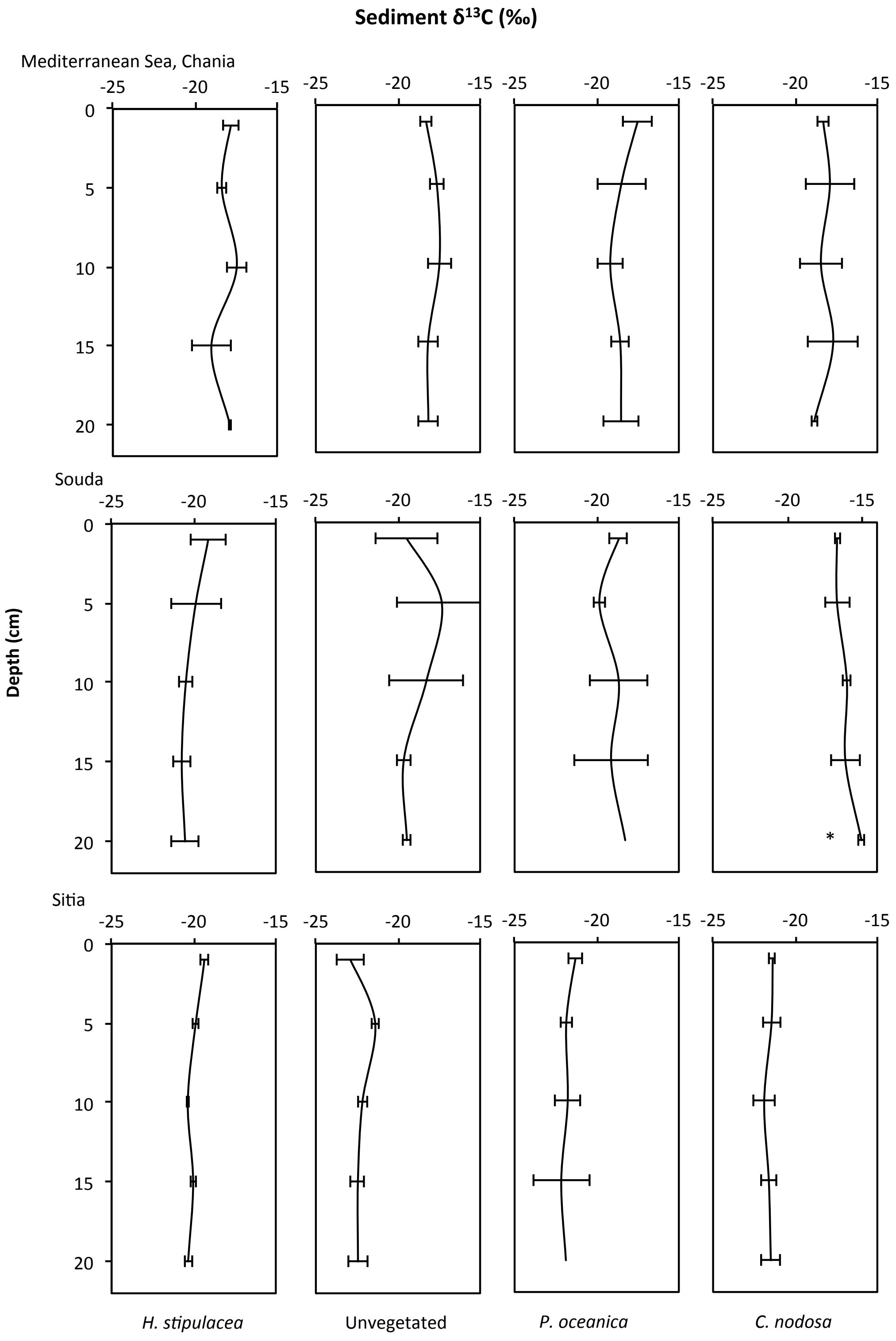

Figure S5. Vertical profile of $\delta^{13}C$ in sediment at each habitat and site. Asterisks indicate significant linear trends with sediment depth.

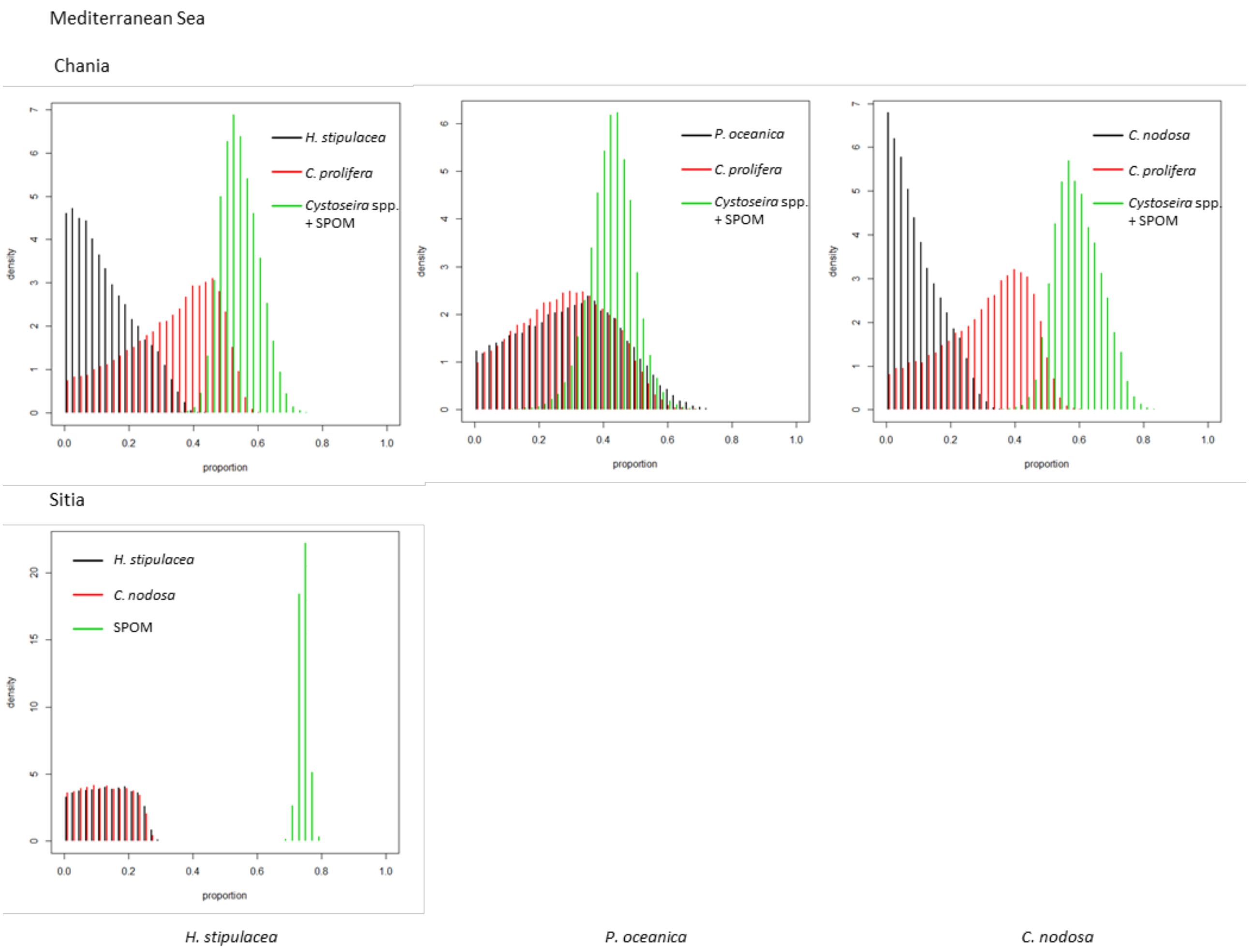

Figure S6. Posterior densities of the mixing model results for Chania and Sitia sites, representing the distribution of possible solutions for all end-members used in each Bayesian mixing model.

Table S1. Grain size (% sand - % silt/ clay) at each site and habitat.

**Biogeographic region**	**Site**	**Habitat**	**Grain size (% Sand - % Silt/ Clay)**
Mediterranean Sea	Chania (35°33'55" N 24°4'48" E)	*H. stipulacea*	90 – 8
		Unvegetated	95 - 4
		P. oceanica	92 - 4
		C. nodosa	98 - 2
	Souda (35°28'17" N 24°8'54" E)	*H. stipulacea*	92 – 8
		Unvegetated	96 - 4
		P. oceanica	87 - 4
		C. nodosa	93 - 6
	Sitia (35°12'26" N 26°0'18" E)	*H. stipulacea*	90 - 8
		Unvegetated	95 - 4
		P. oceanica	91 - 8
		C. nodosa	96 - 3
Red Sea	North Beach (29°32'46" N 34°57'53" E)	*H. stipulacea*	92 - 7 *
	South Beach (29°29´51" N 34°54´45" E)	*H. stipulacea*	54 - 1 (45 gravel) *

Table S2. Mean ± s.d. (‰) of $\delta^{13}C$ in sediment (layer 0-5 cm) and end-members (including the grouped sources) used in the mixing model run for each study site and habitat. References for $\delta^{13}C$ values of end-members taken from literature are also reported.

		End-members						
	**Sediment**	***Halophila stipulacea***	***Posidonia oceanica***	***Cymodocea nodosa***	***Caulerpa prolifera***	***Cystoseira* spp.**	**SPOM**	***Cystoseira* spp. + SPOM**
**Mediterranean Sea**								
Chania - *H. stipulacea*	-18,1 ± 0,5	-9,6 ± 0,7	-		-13,9 ± 0.3[b]	-21,5 ± 1.2[c]	-23,6 ± 0.6[d]	-22,5 ± 1,4
Chania - *P. oceanica*	-18,0 ± 1,2	-	-15,1 ± 0,5		-13,9 ± 0.3[b]	-21,5 ± 1.2[c]	-23,6 ± 0.6[d]	-22,5 ± 1,4
Chania - *C. nodosa*	-18,5 ± 0,3	-	-	-7,4 ± 0,8	-13,9 ± 0.3[b]	-21,5 ± 1.2[c]	-23,6 ± 0.6[d]	-22,5 ± 1,4
Souda - *H. stipulacea*	-19,5 ± 1,3	-7,7 ± 0,8	-	-	-	-	-23,6 ± 0.6[d]	-
Souda - *P. oceanica*	-19,3 ± 0,8	-13,2 ± 0,6	-	-	-	-	-23,6 ± 0.6[d]	-
Souda - *C. nodosa*	-16,7 ± 0,5	-	-	-5,9 ± 0,6	-	-	-23,6 ± 0.6[d]	-
Stia - *H. stipulacea*	-19,6 ± 0,4	-8,5 ± 0,3	-	-8,2 ± 0,4	-	-	-23,6 ± 0.6[d]	-
Stia - *P. oceanica*	-21,6 ± 0,5	-	-15,7 ± 1,0	-	-	-	-23,6 ± 0.6[d]	-
Stia - *C. nodosa*	-21,4 ± 0,4	-	-	-8,2 ± 0,4	-	-	-23,6 ± 0.6[d]	-
**Red Sea**								
North beach - *H. stipulacea*	-18,9 ± 0,1	-7,9 ± 0.1[a]	-	-	-	-	-23,6 ± 0.6[d]	-
South beach - *H. stipulacea*	-21,0 ± 0,2	-7,6 ± 0.1[a]	-	-	-	-	-23,6 ± 0.6[d]	-

[a] M.C. Gambi, G. Winters and S. Vizzini, unpubl. data

[b] Vizzini, unpubl. data

[c] Vizzini and Mazzola, 2009

[d] Vizzini and Mazzola, 2009; Vizzini and others, 2012

Table S3. ANOVA results on structural variables of seagrass meadows.

	Df	Mean Square	F-ratio	P- value	
Two-way ANOVA (Site x Habitat) for Mediterranean Sea region					
Density					
Site	2	45603436	53.16	0.001	***
Species	2	411949618	480.16	< 0.001	***
Site x Species	4	52194931	60.84	< 0.001	***
Residuals	36	857938			
Leaf biomass					
Site	2	12945	21.22	< 0.001	***
Species	2	256764	420.90	< 0.001	***
Site x Species	4	13408	21.98	< 0.001	***
Residuals	18	610			
Rhizome biomass					
Site	2	27709	12.71	< 0.001	***
Species	2	226623	103.94	< 0.001	***
Site x Species	4	27993	12.84	< 0.001	***
Residuals	18	2180			
Root biomass					
Site	2	3308	10.59	0.001	***
Species	2	31563	101.06	< 0.001	***
Site x Species	4	3320	10.63	< 0.001	***
Residuals	18	312			

Table S4. ANOVA results on physiological variables of seagrass meadows.

	Df	Mean Square	F-ratio	P- value	
Two-way ANOVA (Site x Habitat) for Mediterranean Sea region					
Leaf C					
Site	2	61.4	10.22	0.001	**
Species	2	408.3	67.96	< 0.001	***
Site x Species	4	38.2	6.35	0.002	**
Residuals	18	6.0			
Rhizome C					
Site	2	69	1.86	0.185	
Species	2	507	13.62	< 0.001	***
Site x Species	4	137	3.67	0.023	*
Residuals	18	37			
Root C					
Site	2	138	2.95	0.081	
Species	2	337	7.17	0.006	**
Site x Species	4	103	2.20	0.115	
Residuals	16	47			
Leaf N					
Site	2	0.75	6.77	0.006	**
Species	2	0.01	0.06	0.946	
Site x Species	4	0.23	2.05	0.130	
Residuals	18	0.11			
Rhizome N					
Site	2	1.69	4.79	0.024	*
Species	2	0.03	0.08	0.926	
Site x Species	4	0.04	0.10	0.979	
Residuals	16	0.35			
Root N					
Site	2	1.89	8.04	0.004	**
Species	2	0.10	0.43	0.659	
Site x Species	4	0.25	1.07	0.404	
Residuals	16	0.24			
Leaf $\delta^{13}C$					
Site	2	6.49	11.12	0.001	***
Species	2	146	250.92	< 0.001	***
Site x Species	4	1.68	2.88	0.053	
Residuals	18	0.58			

Table S5. Mean DBD, carbon and nitrogen content in the first 20 cm of sediment for each biogeographic region, site and habitat.

Biogeographic region	Site	Species	DBD (g cm^{-3})	Sediment Corg (% DW)	Sediment Cinorg (% DW)	Sediment N (% DW)
Mediterranean Sea	Chania	*H. stipulacea*	0.97 ± 0.25	0.47 ± 0.27	6.05 ± 0.67	0.03 ± 0.02
		Unvegetated	1.16 ± 0.04	0.22 ± 0.02	5.77 ± 0.50	0.02 ± 0.002
		P. oceanica	0.92 ± 0.07	0.95 ± 0.62	13.53 ± 1.10	0.21 ± 0.11
		C. nodosa	1.23 ± 0.03	0.19 ± 0.01	8.38 ± 0.64	0.03 ± 0.01
	Souda	*H. stipulacea*	1.34 ± 0.05	0.25 ± 0.10	8.43 ± 0.20	0.03 ± 0.01
		Unvegetated	1.38 ± 0.03	0.13 ± 0.01	8.34 ± 0.06	0.01 ± 0.001
		P. oceanica	1.31 ± 0.08	0.30 ± 0.03	4.35 ± 0.61	0.03 ± 0.004
		C. nodosa	1.29 ± 0.04	0.21 ± 0.03	9.05 ± 0.15	0.03 ± 0.004
	Sitia	*H. stipulacea*	1.37 ± 0.16	0.25 ± 0.05	2.50 ± 0.17	0.03 ± 0.01
		Unvegetated	1.31 ± 0.07	0.18 ± 0.05	2.26 ± 0.06	0.02 ± 0.002
		P. oceanica	1.27 ± 0.09	0.22 ± 0.04	1.82 ± 0.11	0.02 ± 0.002
		C. nodosa	1.30 ± 0.04	0.13 ± 0.01	2.00 ± 0.24	0.02 ± 0.001
Red Sea	North Beach	*H. stipulacea*	1.26 ± 0.09	0.16 ± 0.03		0.02 ± 0.01
	South Beach	*H. stipulacea*	1.28 ± 0.06	0.14 ± 0.03		0.01 ± 0.01

Table S6. ANOVA results on mean (0 – 20 cm) sediment variables between habitats studied.

	Df	Mean Square	F-ratio	P- value	
Two-way ANOVA (Site x Habitat) for Mediterranean Sea region					
DBD					
Site	2	0.25	298.53	< 0.001	***
Habitat	3	0.03	30.56	< 0.001	***
Site x Habitat	6	0.03	30.45	< 0.001	***
Residuals	24	0.001			
Sediment C_{org}					
Site	2	0.21	19.22	< 0.001	***
Habitat	3	0.17	15.40	< 0.001	***
Site x Habitat	6	0.07	6.72	< 0.001	***
Residuals	24	0.01			
Sediment C_{inorg}					
Site	2	138.17	1745.88	< 0.001	***
Habitat	3	2.75	34.73	< 0.001	***
Site x Habitat	6	25.75	325.42	< 0.001	***
Residuals	24	0.08			
Sediment N					
Site	2	0.01	13.31	< 0.001	***
Habitat	3	0.01	11.64	< 0.001	***
Site x Habitat	6	0.01	9.96	< 0.001	***
Residuals	24	0.001			
One-way ANOVA (Site) for Red Sea region					
DBD					
Site	1	0.001	0.45	0.537	
Residuals	4	0.001			
Sediment C_{org}					
Site	1	0.001	1.41	0.301	
Residuals	4	0.0004			
Sediment N					
Site	1	0.00002	2.67	0.178	
Residuals	4	0.00001			
Two-way ANOVA (Biogeographic region x Site) for *H. stipulacea* habitat					
Sediment C_{org}					
Biogeographic region	1	0.112	220.810	< 0.001	***
Site	3	0.033	64.950	< 0.001	***
Residuals	10	0.001			
Sediment N					
Biogeographic region	1	0.001	180.600	< 0.001	***
Site	3	0.00005	12.360	0.001	**
Residuals	10	0.000004			

References

Vizzini S, Colombo F, Costa V, Mazzola A (2012) Contribution of planktonic and benthic food sources to the diet of the reef-forming vermetid gastropod *Dendropoma petraeum* in the western Mediterranean. Estuar Coast Shelf Sci 96:262–267. doi: 10.1016/j.ecss.2011.11.021

Vizzini S, Mazzola A (2009) Stable isotopes and trophic positions of littoral fishes from a Mediterranean marine protected area. Environ Biol Fishes 84:13–25. doi: 10.1007/s10641-008-9381-3
